# Intact innervation is essential for diet-induced recruitment of brown adipose tissue

**DOI:** 10.1152/ajpendo.00443.2018

**Published:** 2018-12-21

**Authors:** Alexander W. Fischer, Christian Schlein, Barbara Cannon, Joerg Heeren, Jan Nedergaard

**Affiliations:** ^1^Department of Biochemistry and Molecular Cell Biology, University Medical Center Hamburg-Eppendorf, Hamburg, Germany; ^2^Department of Molecular Biosciences, Wenner-Gren Institute, Stockholm University, Stockholm, Sweden

**Keywords:** brown adipose tissue, denervation, diet-induced thermogenesis, obesity, uncoupling protein 1

## Abstract

The possibility that recruitment and activation of brown adipose tissue (BAT) thermogenesis could be beneficial for curtailing obesity development in humans prompts a need for a better understanding of the control of these processes [that are often referred to collectively as diet-induced thermogenesis (DIT)]. Dietary conditions are associated with large changes in blood-borne factors that could be responsible for BAT recruitment, but BAT is also innervated by the sympathetic nervous system. To examine the significance of the innervation for DIT recruitment, we surgically denervated the largest BAT depot, i.e., the interscapular BAT depot in mice and exposed the mice at thermoneutrality to a high-fat diet versus a chow diet. Denervation led to an alteration in feeding pattern but did not lead to enhanced obesity, but obesity was achieved with a lower food intake, as denervation increased metabolic efficiency. Conclusively, denervation totally abolished the diet-induced increase in total UCP1 protein levels observed in the intact mice, whereas basal UCP1 expression was not dependent on innervation. The denervation of interscapular BAT did not discernably hyper-recruit other BAT depots, and no UCP1 protein could be detected in the principally browning-competent inguinal white adipose tissue depot under any of the examined conditions. We conclude that intact innervation is essential for diet-induced thermogenesis and that circulating factors cannot by themselves initiate recruitment of brown adipose tissue under obesogenic conditions. Therefore, the processes that link food intake and energy storage to activation of the nervous system are those of significance for the further understanding of diet-induced thermogenesis.

## INTRODUCTION

An important incentive for the present interest in brown adipose tissue as such, and in white adipose tissue browning as well, is undoubtedly the expectation that recruitment and activation of the thermogenic potential of these tissues could help in curbing the increase in obesity presently encountered worldwide. Therefore, it is of considerable interest to understand the physiological pathways that augment the recruitment processes in these tissues when individuals are exposed to obesogenic conditions. Such pathways could be of hormonal nature, i.e., food-related, blood-borne substances that would directly interact with the tissues - or they could be mediated via the innervation of the tissues.

In this respect, it is relevant for the clarification of the pathways involved that the thermogenic capacity of brown adipose tissue (BAT) is involved in two physiological, homeostatic processes: both the maintenance of body weight during overfeeding as referred to here (often termed “diet-induced thermogenesis”) and the maintenance of body temperature in the cold (termed “classical non-shivering thermogenesis”).

Concerning the classical nonshivering thermogenesis, including both acute activation of BAT in the cold and the recruitment of the tissue that takes place during prolonged exposure to cold, numerous studies have demonstrated an important or even essential role of the nerves innervating the tissue in this process. Thus, surgical denervation of the tissue leads to the absence of acute cold-induced activation of the tissue (31, 34, 42, 46, 60, 71, 74) as well as to the absence of the characteristics of the recruited tissue, such as the cold-induced expression of the unique heat-producing protein located in the inner mitochondrial membrane, i.e., uncoupling protein 1 (UCP1) (17, 18, 21, 45), the enhanced brown adipocyte differentiation (17, 45, 60), and the increased cell proliferation in the tissue (34, 55).

Concerning diet-induced thermogenesis, the situation is more complex. Although vaguely defined, diet-induced thermogenesis (DIT) is generally considered to be composed of two components, an obligatory part, i.e., the heat necessarily released during digestion and processing of the food, and a facultative part that involves recruitment of thermogenic mechanisms and increases in energy expenditure (85). It is this latter part that is of relevance to the question raised here. Rothwell and Stock (61) found in 1979 that facultative DIT was mediated, at least partly, through BAT-mediated thermogenesis. Although the existence of facultative DIT has been questioned (49, 52), several studies have reported that the recruitment state of BAT is increased in animals fed energy-rich diets (3, 20, 33, 48, 61, 63, 78, 85), and others have found that UCP1 deficiency increases susceptibility for the development of obesity (20, 48, 63, 89) and is associated with reduced obesity- and meal-induced increases in energy expenditure (85). Thus, brown-fat-mediated DIT seems to exist.

The issue is thus whether the innervation is as important for DIT as it is for classical nonshivering thermogenesis or whether humoral pathways would be of major importance here. Concerning DIT, the existing evidence from surgical denervation has addressed primarily the acute activity of the tissue; denervation of BAT led to a blunted increase in mitochondrial GDP binding in rats fed an energy-rich diet (60), and this lower “unmasking” is normally interpreted as an indication of an acutely lower activity of UCP1 in these animals. Additionally, the increase in total protein content and mitochondrial content found in rats fed an energy-rich diet was blunted in the denervated rats (60).

Thus, whereas the evidence for sympathetically mediated control of both acute BAT activity and BAT recruitment in the cold is strong, there is no parallel evidence for this control during DIT. Therefore, there is good reason to examine the involvement of the innervation of BAT in the regulation of DIT. This is especially true because in recent years there has been a large interest in the possibility that nonneuronal, i.e., humoral, factors may be able to activate and recruit BAT (5, 19, 29, 82, 83, 90). These factors include circulating factors, such as FGF21 and cardiac natriuretic peptides — or metabolites such as bile acids, etc. Also, an involvement of macrophages as sites of norepinephrine synthesis in the tissue has been suggested (56), but present evidence does not favor such a mechanism (28). That DIT could be largely or totally induced via circulating factors would indeed be reasonable, considering the many circulating factors that are associated with food intake and food energy status (leptin, ghrelin, cholecystokinin, insulin, bile acids, acetate, etc.). Therefore, in the current study, we examined the need for the nervous supply to BAT, particularly for the adaptive response of the tissue to an overfeeding regime. We find that, similar to the case for cold-induced thermogenesis, the recruitment of the tissue during DIT is fully dependent upon an intact nervous supply of the tissue.

## METHODS

### 

#### Animal studies.

For the experiments, male C57BL/6J mice aged ∼12 wk, raised at 22°C, and bred at the local animal breeding facility at University Medical Center Hamburg-Eppendorf were used. All mouse experiments were approved by the Animal Welfare Officers of the University Medical Center Hamburg-Eppendorf (UKE) and Behörde für Gesundheit und Verbraucherschutz Hamburg. The mice were housed on a 12:12-h light-dark cycle with ad libitum access to food and water. The mice were fed a chow diet (P1324; Altromin) or high-fat diet (HFD; EF Bio-Serv no. F3282 mod., 60% calories from fat; Sniff). The mice were single-caged before the surgical procedure. All mice had access to bedding, paper nesting material, and paper rolls for nest building. Throughout the experiments, the mice were housed at 30°C.

#### Denervation of interscapular BAT.

**Fig. 1.**
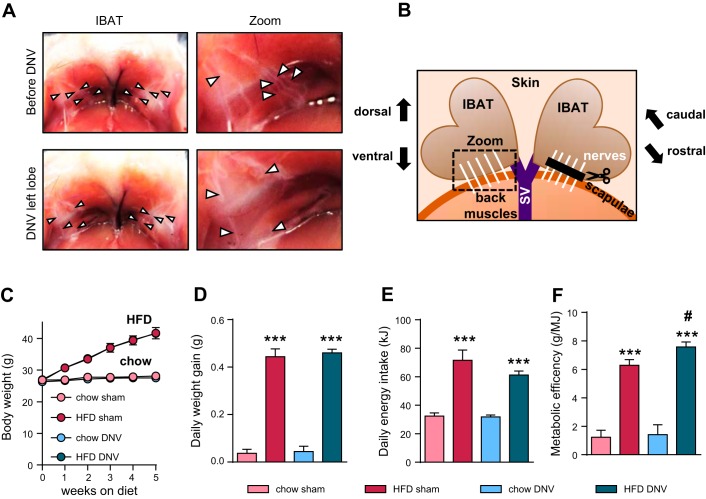
Effects of interscapular brown adipose (IBAT) tissue denervation on weight gain. IBAT of male C57BL/6J mice was surgically denervated. After a 4-wk recovery period at 30°C, the mice were fed chow or a high-fat diet for 6 wk at 30°C. *A*, *top left*: view on the back of a mouse, looking in direction of the head. IBAT has been detached from back muscles and lifted up to expose the nerves and the ventral side of the IBAT lobes. The picture shows the nerve fiber bundles innervating each lobe of the tissue (arrowheads point toward the area of innervation). In the middle of the image, Sulzer’s vein is visible. *A, top right*: the individual fiber bundles can be seen (here, arrowheads point toward individual nerve bundles). For denervation (DNV), the fibers were cut at 2 different locations, and the intermediate piece was removed to prevent regrowth of the fibers. *A*, *bottom left*: shows IBAT morphology; the left nerves have been cut (arrowheads), and the nerves innervating the right lobe are still intact here (arrowheads), but in the experimental animals both nerve bundles were cut. *A, bottom right*: cut nerves, lacking the intermediate pieces, as indicated by arrows pointing toward the cut fibers. *B*: schematic representation of the anatomic location of the nerves and the IBAT lobes shown in the images in *A*. The 2 heart-shaped IBAT lobes are also here seen from below (from the ventral side), and innervating nerves (white) enter and the Sulzer’s vein (SV) leaves the tissue from below (the scapulae region). In the mouse, the 2 lobes are usually attached to the back muscles shown at the bottom of the picture. The dashed box indicates the area shown in the zoomed pictures in *A*, and the scissors and black bar at the right nerve bundles indicate the denervation process. Note that in the present study, denervation was performed on both lobes. *C*: body weight curve of IBAT sham or IBAT-denervated (DNV) animals on chow or high-fat diet (HFD). Only weight gain during the first 5 wk is shown, since indirect calorimetry was performed afterward. Note that the sham and DNV chow curves, as well as the sham and DNV HFD curves, fully overlap; thus, the blue points are not visible. Also note that the standard errors on some points were smaller than the size of the symbols. *D*: mean daily weight gain of the 4 treatment groups during *weeks 2–5* of the feeding study. *E*: mean daily energy intake of the 4 treatment groups during *weeks 2–5* of the feeding study. *F*: metabolic efficiency, expressed for each mouse as gram of body weight gained per MJ food energy during *weeks 2–5* of the feeding study. All values are means ± SE. ***P ≤ 0.001 between diets and #*P* ≤ 0.05 between surgical intervention groups; *n* = 5.

Denervation of interscapular BAT (IBAT) was performed as described by Vaughan et al. (80). Briefly, mice were anesthetized using isoflurane inhalation anesthesia, and the area above the interscapular region of the mice was shaved and sterilized with 80% ethanol. The skin was opened in a ∼1-cm incision and the brown adipose tissue prepared by detaching it from the underlying muscle layer. The nerve fibers innervating each BAT lobe were exposed and cut, or only exposed and touched in the sham groups; see also Fig. 1, *A* and *B*. After the procedure, the fat pads were rinsed with sterile 0.9% NaCl solution, the incision was closed using Ethilon II 4-0 suture (Ethicon Endo-Surgery), and the animals were placed in clean cages and allowed to recover for ∼4 wk at 30°C. Starting 1 day before the surgery and during a recovery period of 4 days, the mice received analgesia (0.2 mg/kg Meloxicam via food) and were additionally injected with carprofen immediately before the surgery (5 mg/kg carprofen ip).

#### Experimental setting.

After the recovery period, the mice remained at 30°C. The mice were fed the respective diet for 6 wk, and body weight and food intake were measured weekly. At the end of the study, mice were fasted for 4 h and anesthetized with ketamin/Rompun injection anesthesia (180 mg/kg ketamin and 24 mg/kg Rompun). Mice were then perfused transcardially with phosphate-buffered saline containing 10 U/ml heparin, the indicated organs were collected, and the adipose tissues were immediately snap-frozen in liquid nitrogen, or immersion-fixed in 3.6% formalin in phosphate-buffered saline for immunohistochemistry.

#### Calculation of energy intake.

For calculation of energy intake, weekly food intake was multiplied with the metabolizable energy content of the diets, as stated by the manufacturers: chow diet 11.92 kJ/g and HFD 22.7 kJ/g. This calculation was performed for weekly food intake as well as food intake during indirect calorimetry measurements.

#### Indirect calorimetry.

After a feeding period of 5–6 wk, the mice were transferred to Tecniplast green line IVC cages and allowed to adapt to the new cage overnight at 30°C. After this adaptation, 24-h metabolic measurements were performed. Measurements were performed using a TSE PhenoMaster System (TSE Systems) in a temperature-controlled chamber at 30°C. O_2_ consumption and CO_2_ production, as well as food and water intake, were measured every 15 min. Food and water consumption were measured as changes in food weight within the 15-min measurement interval, with corrections for movements caused by the mouse accessing the food tray. Energy expenditure was calculated using a modified Weir equation (24–26, 87):

$$\begin{array}{l} \text{Energy expenditure [W]}\;=\;0.2716\;\frac{W\times \min}{\text{ml}}\\ \;\times {\text{V(O}}_{\text{2}}{\text{)}}_{\text{consumed}}\;[\frac{\text{ml}}{\min}]+\;0.07616\;\frac{W\times \min}{\text{ml}}\\ \;\times {\text{V(CO}}_{\text{2}}{\text{)}}_{\text{produced}}\;[\frac{\text{ml}}{\min}] \end{array}$$

Respiratory quotient was calculated as

$$\text{RQ}=\frac{{\text{V(CO}}_{\text{2}}{\text{)}}_{\text{produced}}}{{\text{V(O}}_{\text{2}}{\text{)}}_{\text{consumed}}}.$$

To quantify the contribution of HFD feeding to whole body metabolism, the energy expenditure of chow and HFD-fed mice was divided by the average energy expenditure of chow-fed, sham-operated mice (see Fig. 3, *C*–*E*). After the calorimetric measurements, the mice were transferred back to standard cages and remained there until euthanasia.

#### Western blotting.

For Western blotting, organs were dissected and snap-frozen immediately. Tissues were homogenized in 10:1 (vol/wt) RIPA buffer (50 mM Tris·HCl, pH 7.4, 5 mM EDTA, 150 mM sodium chloride, 1 mM sodium pyrophosphate, 1 mM sodium fluoride, 1 mM sodium orthovanadate, and 1% NP-40) supplemented with cOmplete Mini Protease Inhibitor Cocktail Tablets (Roche) in a Tissue Lyser (Qiagen) at 20 Hz for 2 × 3 min. The homogenate was centrifuged at 14,000 *g* for 10 min at 4°C, and the supernatant was removed using a syringe and a 27 G needle (to avoid contamination from the upper fat layer). Protein concentration in the supernatant was determined using the Lowry method.

The protein lysates were mixed with NuPAGE LDS Sample Buffer and NuPAGE Sample Reducing Agent (Invitrogen), and 20 µg of total protein was separated in 10% Tris-glycine SDS-PAGE. Transfer to nitrocellulose membranes (GE) was performed in a wet blotting system in blotting buffer [20 mM Tris, 150 mM glycine, 20% (vol/vol) methanol] at 4°C for 2 h at 400 mA or overnight at 200 mA. Membranes were stained with Ponceau Red (Sigma), cut, and blocked for 1 h in 5% milk in TBS-T [20 mM Tris, 150 mM sodium chloride, 0.1% (vol/vol) Tween 20]. Membranes were incubated overnight at 4°C with the corresponding primary antibodies in 5% BSA (Sigma) in TBS-T. After washing 3 × 10 min in TBS-T, membranes were incubated for 1 h at room temperature with goat-anti rabbit HRP-conjugated secondary antibody (Jackson Immunoresearch no. 111-035-144; 1:5,000) in 5% milk in TBS-T. After an additional 3 × 10 min washing in TBS-T, detection was performed using SuperSignal West Femto Maximum Sensitivity Substrate (Thermo). For detection, an Amersham Imager 600 (GE) was used, and quantification was performed using Li-Cor Image Studio Lite (Li-Cor).

The following primary antibodies were used: anti-UCP1 (rabbit polyclonal, noncommercial, against the COOH-terminal decapeptide, 1:12,500); anti-tyrosine hydroxylase (rabbit monoclonal, Abcam ab137869, 1:5,000), anti-γ-tubulin (rabbit monoclonal, Abcam ab179503, 1:2,000).

#### RNA isolation and quantitative real-time PCR.

Tissue samples (50–100 mg) were homogenized in 1 ml TriFast (Peqlab) in a Tissue Lyser (Qiagen) at 20 Hz for 2 × 3 min; 250 μl chloroform was added, and samples were vortexed and centrifuged for 15 min at 14,000 *g*. The supernatant was mixed with 70% ethanol (1/3 vol/vol) and RNA was extracted with a commercial RNA extraction kit (Nucleo Spin RNA kit; Macherey-Nagel) according to the manufacturer’s instructions. RNA purity and concentration were determined using the NanoDrop ND-1000 system (PeqLab). cDNA synthesis was performed with 400 ng of RNA using High Capacity cDNA Reverse Transcription Kit (Applied Biosystems) with random hexamer primers, following the manufacturer’s instructions. Relative mRNA levels were determined by quantitative RT-PCR using the TaqMan Gene Expression Assay Technique and the 7900HT Fast Real-Time PCR System (Applied Biosystems). The following assays were used: mTbp (Mm00446973_m1), mUcp1 (Mm00494069_m1), mAdrb3 (Mm00442669_m1), and mDio2 (Mm00515664_m1). Gene expression was normalized to the housekeeper TATA-box binding protein (mTbp) using the ΔΔC_T_ method.

#### Histology and immunofluorescence staining.

For tissue staining, a piece of IBAT (1/2 lobe) was immersion-fixed in 3.6% formalin in phosphate-buffered saline at 4°C for several days. The tissues were automatically dehydrated and embedded in paraffin in an Autotechnikon apparatus (Leica). Paraffin blocks were cut into 4-µm sections on a Leica Microtome and dried overnight at 42°C. The sections were rehydrated with the following steps: 2 × 10 min in Roti-Histol (Roth), 2 × 5 min in 100% ethanol, 5 min in 96% ethanol, 5 min in 70% ethanol, 5 min in 50% ethanol, and 2 × 5 min in dH_2_O. For hematoxylin and eosin staining, sections were then incubated for 5 min in Mayer’s Hematoxylin (Sigma), blued under running tap water for 10 min, stained with 5% Eosin Y for 30 s, rinsed in dH_2_O, and dehydrated in 70% ethanol, 96% ethanol, 2× 100% ethanol, and 2× Roti-Histol. Mounting was performed using Eukitt mounting media (O. Kindler).

For immunostaining, sections were rehydrated as described above. Then, antigen retrieval was performed by boiling the slides for 30 min in citrate buffer [10 mM trisodium citrate (Sigma), pH 6]. Slides were cooled to room temperature, and background fluorescence was blocked by incubating the slides for 5 min in 0.1% Sudan black B (Sigma) in 70% ethanol. Slides were rinsed with PBS, and blocking was performed using 3% BSA (Sigma) and 0.1% Triton X100 (Sigma) in PBS (Gibco) for 1 h at room temperature. Primary antibody incubation was performed overnight at 4°C in a humid incubation chamber. The following antibodies, diluted in 3% BSA in PBS (Gibco), were used: anti-tyrosine-hydroxylase (rabbit monoclonal, ab137869, 1:200; Abcam) and anti-perilipin1 (goat polyclonal, ab61682, 1:1,000; Abcam). After incubation, slides were washed 3 × 10 min in TBS-T and incubated for 1 h at room temperature in secondary antibodies diluted in 3% BSA in PBS. The following secondary antibodies were used: Cy2-donkey-anti-goat (705-225-147; 1:500; Jackson Immunoresearch) and Cy3-donkey-anti-rabbit (711-165-152; 1:500; Jackson Immunoresearch). After incubation, slides were washed for 3 × 10 min in TBS-T (in the dark) and mounted using Roti-Mount FluorCare DAPI mounting media (Roth). Imaging was performed on a NikonA1 Ti microscope equipped with a DS-Fi2-U3 camera for bright-field imaging. Fluorescence microscopy was performed using the NikonA1 Ti confocal laser scanning microscope with 405-, 488-, and 561-nm lasers and a galvano scanner head (detector cubes: 425–475, 500–550, and 570–620 nm). Image processing was performed using NikonAR software.

#### Data processing and statistics.

Data analysis was performed in Microsoft Excel 2016 and GraphPad Prism 6. For statistical analysis, Student’s *t*-test was used, and *P* ≤ 0.05 was considered to be statistically significant (in the figures, *P ≤ 0.05, **P ≤ 0.01 and ***P ≤ 0.001 for effects of high-fat vs. chow, and #*P* ≤ 0.05, ##*P* ≤ 0.01 and ###*P* ≤ 0.001 for effects of denervation vs. sham.

## RESULTS

### 

#### Effects of IBAT denervation on weight gain.

Feeding mice a calorie-rich obesogenic diet, especially under conditions of low thermogenic stimulus (i.e., at thermoneutrality), leads to a gradual recruitment of BAT, as indicated by increased total UCP1 levels and an increased thermogenic response to norepinephrine (3, 20, 33, 48, 61–63, 81, 85). To examine the necessity of tissue innervation for the control of the recruitment response, we denervated the interscapular brown adipose tissue depot (IBAT) of wild-type (WT) mice and fed them either chow or high-fat diet under thermoneutral conditions. It should be noted that it was only the interscapular (IBAT) depot that was denervated. IBAT represents only a fraction of the total BAT depots, with its estimated contribution to thermogenic potential ranging from 70 (1, 2) to 25% (72), depending on the methodology used to determine the contribution. Correspondingly, in mice at 30°C, ∼45% of total UCP1 mRNA is found in IBAT, whereas other BAT depots [subscapular/axillary BAT and cervical BAT, as well as periaortic and perirenal BAT depots (6, 44)] account for ∼50% and brite/beige depots for ∼5% of total UCP1 mRNA (14, 44). Thus, IBAT denervation would not be expected to be equivalent to total loss of BAT activity, and systemic effects of the denervation may thus be reduced by the still intact (and possibly enhanced) recruitment in other depots.

Each lobe of IBAT is innervated by five nerve bundles (Fig. 1, *A* and *B*), as indicated by arrowheads in Fig. 1*A*. These nerves were either surgically exposed (sham) or cut (denervation), as indicated by arrowheads in Fig. 1*A*. Denervation was performed on both IBAT lobes. The schematic localization of the nerves in the BAT depot is depicted in Fig. 1*B*. The efficiency of the denervation was confirmed at the end of the experiment (see below).

The body weight of chow-fed mice was stable and was not affected by denervation (Fig. 1*C*). Feeding the mice a high-fat diet resulted, as expected, in weight gain (Fig. 1, *C* and *D*). This weight gain was also not affected by IBAT denervation (Fig. 1, *C* and *D*). The mice on high-fat diet had significantly higher energy intake (Fig. 1*E*), which was statistically similar (but tended to be slightly lower) in mice with denervated IBAT (Fig. 1*E* and Table 1). Thus, equal weight gain was obtained with seemingly slightly lower energy intake in the denervated mice, which formally resulted in a significantly higher metabolic efficiency; that is to say, the fraction of ingested calories that was stored was higher in the IBAT-denervated mice than in the sham mice (Fig. 1*F*). These data thus indicate that the absence of innervation of IBAT significantly affected energy balance in the mice, and although the mice did not become more obese, they became equally obese with a lower caloric intake. The mice thus apparently reacted to the denervation by adjusting food intake.

**Table 1.** Quantification of the metabolic parameters assessed during indirect calorimetry

	Energy Intake, kJ			RQ			EE, W		
	Day	Night	Diurnal	Day	Night	Diurnal	Day	Night	Diurnal
Sham chow	7 ± 1	21 ± 2	28 ± 36	0.89 ± 0.02	0.96 ± 0.03	0.93 ± 0.02	0.26 ± 0.02	0.39 ± 0.03	0.32 ± 0.02
Sham HFD	20 ± 2***	35 ± 2***	54 ± 2***	0.83 ± 0.01*	0.83 ± 0.02**	0.83 ± 0.01**	0.35 ± 0.01**	0.47 ± 0.02†	0.41 ± 0.02*
DNV chow	7 ± 1	23 ± 3	30 ± 3	0.91 ± 0.03	0.94 ± 0.03	0.92 ± 0.03	0.28 ± 0.03	0.43 ± 0.03	0.36 ± 0.03
DNV HFD	15 ± 2*	33 ± 2*	48 ± 3**#	0.83 ± 0.01*	0.82 ± 0.02*	0.82 ± 0.02*	0.33 ± 0.02	0.46 ± 0.02	0.40 ± 0.02

**Fig. 2.**
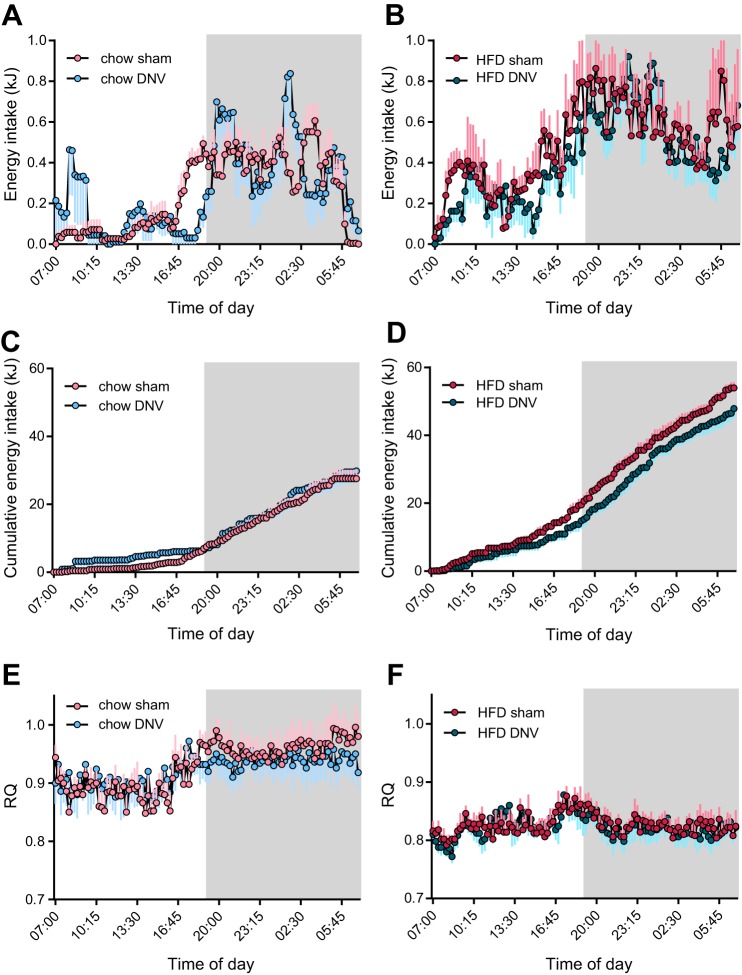
Metabolic effects of interscapular brown adipose (IBAT) denervation. After 5 wk of feeding, the mice shown in Fig. 1 were subjected to indirect calorimetry analysis to examine the effects of IBAT denervation on whole body metabolism. *A*: diurnal pattern of energy intake in the chow-fed animals during the course of the calorimetry measurements. *B*: diurnal pattern of energy intake in the high-fat diet (HFD)-fed animals during the course of the calorimetry measurements. *C*: cumulative energy intake in the chow-fed animals during the calorimetry study. *D*: cumulative energy intake in the HFD-fed animals during the calorimetry study. *E*: respiratory quotient (RQ) of the chow-fed animals with either sham operation or IBAT denervation (DNV). *F*: respiratory quotient (RQ) of the HFD-fed animals with either sham operation or IBAT denervation. All values are means and SE; *n* = 5. Mean day/night and diurnal values for RQ and energy intake are shown in Table 1. Data points for food intake and RQ were recorded every 15 min. The data in *A* and *B* were smoothened over 5 time points.

**Fig. 3.**
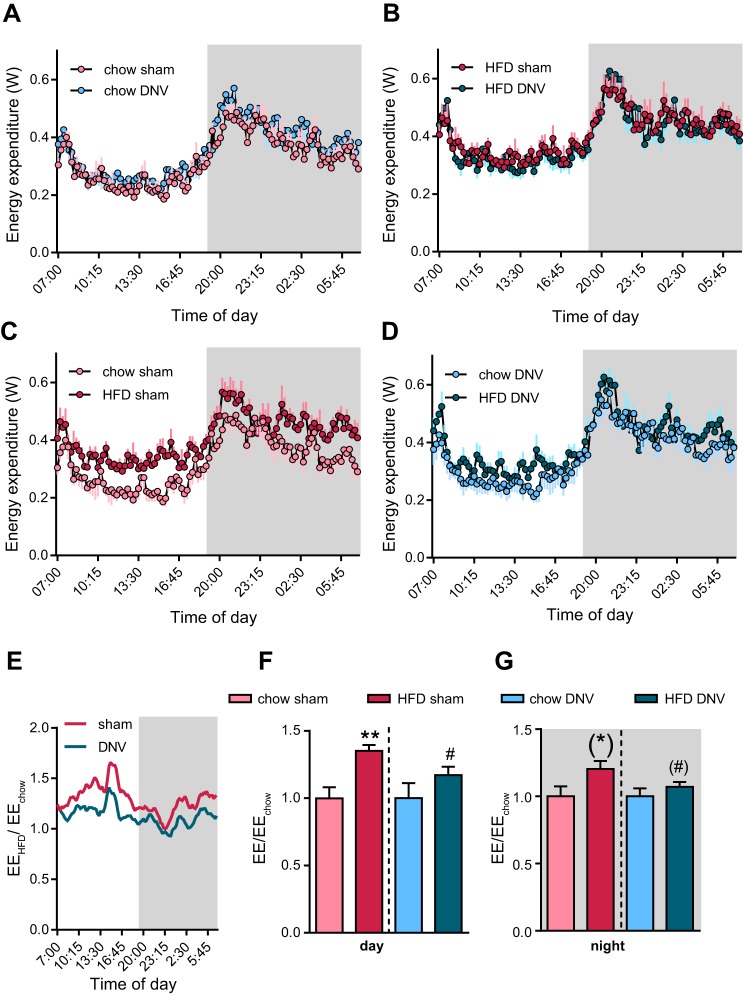
Effects of interscapular brown adipose (IBAT) denervation on the calorigenic response to diets. Energy expenditure (EE) during the experiment shown in Fig. 2. *A*: diurnal pattern of energy expenditure (in W) of the chow-fed animals with either sham operation or IBAT denervation. *B*: diurnal pattern of energy expenditure (in W) of the high-fat diet (HFD)-fed animals with either sham operation or IBAT denervation. *C*: diurnal energy expenditure pattern of chow- and HFD-fed, sham-operated mice. *D*: diurnal energy expenditure pattern of chow and HFD-fed, IBAT-denervated (DNV) mice. Note that the data in *C* and *D* are the same as in *A* and *B* but combined differently to show the effect of diet within the same intervention group. *E*: fold change of EE in HFD-fed animals over respective chow-fed controls. The data were smoothened over 5 time points. *F*: quantification of fold change of daytime EE in sham-operated and IBAT-denervated animals over mean of respective chow-fed controls (dashed line indicates normalization to different control values). *G*: quantification of fold change of nighttime EE in sham-operated and IBAT-denervated animals over mean of respective chow-fed controls (dashed line indicates normalization to different control groups). (#)Induction of energy expenditure in HFD-fed animals trended to be lower (*P* = 0.09) in IBAT-denervated mice (dark blue) than in sham-operated mice (dark red). Data points were recorded every 15 min. All values are means and SE. Note that in *E*, mean values were compared and thus no standard deviation could be calculated. (*)*P* ≤ 0.1 and **P ≤ 0.01 between diets, #*P* ≤ 0.05 between surgical intervention groups; *n* = 5. Mean day/night and diurnal values for EE are shown in Table 1.

Values are means  ± SE. DNV, denervation; EE, energy expenditure; HFD, high-fat diet; RQ, respiratory quotient. Day-night and diurnal levels of energy intake, RQ, and EE are shown. This is the quantification of the data shown in Figs. 2 and 3.

† *P* ≤ 0.1,

* *P* ≤ 0.05,

** *P* ≤ 0.01,

and

*** *P* ≤ 0.001

between diets, and

# *P* ≤ 0.1

between treatments; *n* = 5.

#### Metabolic effects of IBAT denervation.

Diet-induced recruitment of BAT leads not only to an elevated response to the injection of thermogenic agents such as norepinephrine (20, 61) but also to increased whole body energy expenditure (85), an effect that is dependent on the presence of UCP1 (85). Therefore, we examined whether elimination of IBAT sympathetic innervation had effects on whole body energy expenditure. However, as stated above, it has to be remembered that IBAT is only one of the BAT depots in mice, and the innervation to the other depots is still intact in this setting.

After a feeding period of 5–6 wk, the mice were thus subjected to 24-h indirect calorimetry at 30°C, and food and water intake as well as energy expenditure were continuously monitored during these 24 h. Chow-fed mice ate mainly during the dark phase (Fig. 2*A*), whereas mice on high-fat diet consumed food throughout the entire day, with the intake during the dark phase being higher (Fig. 2*B*). This was also reflected by different cumulative energy intake in these groups (Fig. 2, *C* and *D*), with the total energy intake of the HFD-fed mice during the 24-h period being markedly higher than that of the chow-fed mice (Fig. 2, *C* and *D* and Table 1). The IBAT-denervated groups showed a shifted feeding pattern on both diets (Fig. 2, *A*–*D*), with a delayed induction of feeding at the end of the light phase and increased feeding at the beginning of the light phase in chow-fed IBAT-denervated animals (Fig. 2, *A* and *C*). In addition, the IBAT-denervated mice on both diets showed a lower meal frequency during the light phase (not shown). This was accompanied by a reduced cumulative food intake in the mice on high-fat diet (Fig. 2*D* and Table 1). This marked effect of IBAT denervation on feeding behavior in mice is not immediately understandable but may involve loops between IBAT activity and hypothalamic centers.

The respiratory quotient, a readout of metabolic fuel preference, was not different between IBAT-denervated or sham-operated mice on either diet (Fig. 2, *E* and *F*). Chow-fed animals showed a rhythmicity of respiratory quotient (RQ) levels, the RQ being ≈0.9 during daytime, indicating somewhat enhanced fat oxidation during the phases of lower food intake, and ≈0.95 during nighttime, indicating high carbohydrate oxidation during the active phase (Fig. 2, *E* and *F*, and Table 1), in accordance with the diet composition. High-fat diet-fed animals showed almost no rhythmicity, displaying a constant RQ of ≈0.8 (Fig. 2, *E* and *F*, and Table 1). The calculated RQ value for the high-fat diet itself was 0.82 (assuming RQ of 0.7 for fat, 0.8 for protein, and 1 for carbohydrate oxidation), and the RQ thus directly reflected the food composition in these mice.

Concerning energy expenditure, all groups showed the expected diurnal rhythmicity, with nighttime levels being higher than daytime levels, reflecting the active and resting phases of the animals (Fig. 3, *A* and *B*, and Table 1). No statistically significant differences in energy expenditure were observed between the sham-operated and denervated groups on the chow diet, nor were any marked differences seen between the two groups on HFD. However, a comparison of the two diets in the sham-operated groups demonstrated a significantly elevated energy expenditure in the HFD-fed sham-operated group (Fig. 3*C* and Table 1). This is in principle in line with previous observations (85), where the elevated metabolism in HFD-fed animals was shown to be UCP1-dependent. In contrast, in IBAT-denervated mice, HFD feeding did not lead to significantly elevated levels of energy expenditure (Fig. 3*D* and Table 1). This was due mainly to slightly elevated baseline levels in the denervated chow-fed animals (Fig. 3*A* and Table 1). When the effect of HFD (fold increase over chow) was plotted (Fig. 3*E*), it could be observed that sham-operated mice always had a higher energy expenditure than did the denervated mice. IBAT-denervated mice showed a significantly reduced effect of high-fat diet during daytime (Fig. 3*F*) and a slightly diminished effect during nighttime (Fig. 3*G*). This is in principle in line with the elevated metabolic efficiency observed over a longer time in these mice (Fig. 1*F*).

#### IBAT structure is affected by denervation.

**Fig. 4.**
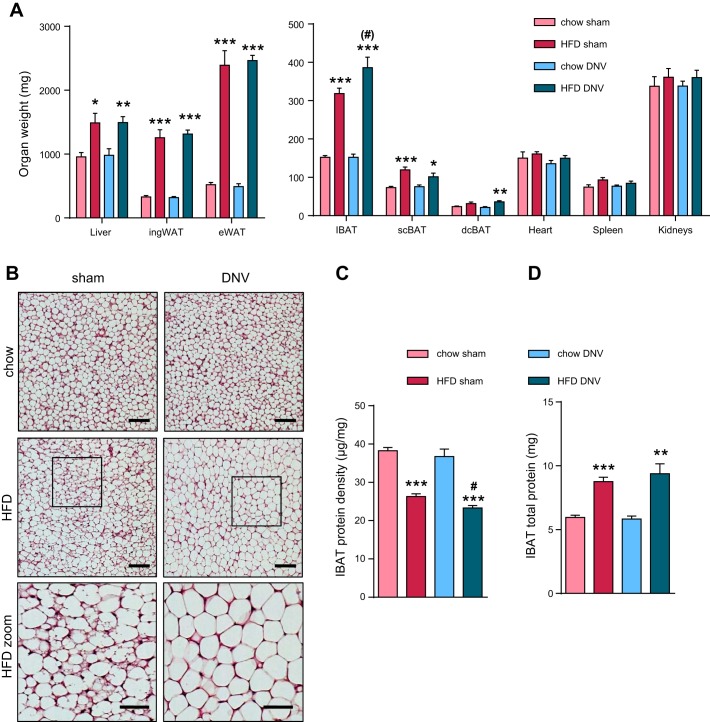
Effects of interscapular brown adipose (IBAT) denervation on organ weight and brown adipose tissue (BAT) morphology. Organ weights and general parameters of BAT function were assessed in the different groups of mice. *A*: organ weights. *B*: representative pictures of hematoxylin and eosin-stained sections of IBAT of chow- and HFD-fed mice after sham operation or IBAT denervation (DNV). Box indicates area shown in the magnified images below. Scale bar, 100 µm. Images at *bottom* show higher magnification of IBAT of sham-operated and iBAT-denervated, HFD-fed mice, illustrating the appearance of multilocular adipocytes in IBAT of sham-operated but not in IBAT-denervated mice. Scale bar, 50 µm. *C*: IBAT protein density (µg protein/mg tissue) in the respective groups. *D*: IBAT total protein content per depot in the respective groups (calculated as protein density multiplied by tissue weight). All values are means ± SE. *P ≤ 0.05, **P ≤ 0.01, and ***P ≤ 0.001 between diets, (#)*P* ≤ 0.1 and #*P* ≤ 0.05 between surgical intervention groups; *n* = 5. dcBAT, dorsocervical brown adipose tissue; eWAT, epididymal white adipose tissue; ingWAT, inguinal white adipose tissue; scBAT, subscapular (also known as axillary) brown adipose tissue.

After the end of the experiment, several organs were dissected out. As mentioned, IBAT denervation did not lead to increased body weight (Fig. 1, *C* and *D*). This was reflected in similar weights of liver and white adipose tissue in sham-operated and IBAT-denervated mice both on chow and HFD (Fig. 4*A*). However, IBAT weight tended to be increased in IBAT-denervated mice fed HFD but not in chow-fed animals (Fig. 4*A*). In line with this observation, histological analysis revealed no effect of IBAT denervation on IBAT morphology in mice fed chow diet (Fig. 4*B*; note that all animals had been acclimated to thermoneutrality and thus display lipid-filled brown adipocytes). HFD feeding induced the appearance of islands of multilocular cells in the IBAT depot of sham-operated mice (Fig. 4*B*), in agreement with the higher tissue activation observed under such conditions (85). This effect was not observed in denervated IBAT, and the lipid droplets appeared marginally larger (Fig. 4*B*). In line with this histological analysis, protein density (µg protein/mg tissue) was similar in sham-operated and denervated chow-fed mice (Fig. 4*C*). Because of the increased lipid accumulation under obesogenic conditions, HFD-feeding led to a reduced protein density, and denervation of IBAT led to a somewhat greater reduction in protein density on HFD, in agreement with the histological observations (Fig. 4*C*). However, importantly, in both treatment groups, HFD feeding robustly increased the total protein content of the tissue (Fig. 4*D*), which may be secondary to increased lipid deposition.

#### Denervation of IBAT effectively reduces tyrosine hydroxylase levels.

To examine the effectiveness of the denervation procedure, we performed Western blot analysis of the IBAT samples obtained at the end of the experiment. Tyrosine hydroxylase (TH) is the rate-limiting enzyme of catecholamine synthesis, catalyzing the generation of the norepinephrine precursor L-DOPA from l-tyrosine (75). The gene is transcribed and the protein is synthesized in the neuronal body and is then transported to the nerve endings.

**Fig. 5.**
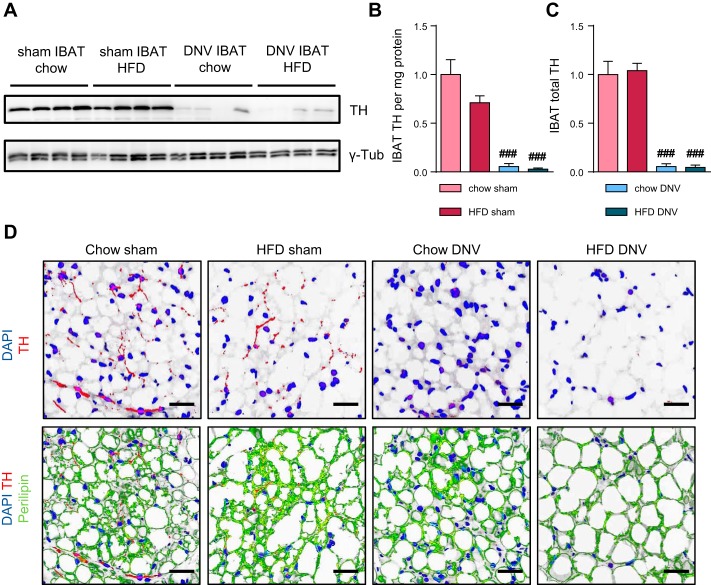
Interscapular brown adipose (IBAT) denervation (DNV) efficiency *A*: Western blot of 20 µg of IBAT protein from chow- and HFD-fed animals that either had undergone sham operation or IBAT DNV. Note that because of the close molecular weights of tyrosine hydroxylase (TH; ∼58 kDa) and γ-tubulin [γ-tub (as loading control); ∼50 kDa], the loading control was run on a different gel but on the same samples. *B*: quantification of the blot shown in A, representing the concentration of TH/mg protein. Data in *B* and *C* are normalized to the mean of chow-fed, sham-operated controls. *C*: total tissue TH content, calculated as TH/mg protein multiplied by total protein content (*B* and *D*, respectively) and normalized to the mean of chow-fed, sham-operated controls. *D*: representative pictures of paraffin sections of IBAT from chow- and HFD-fed animals that had undergone either sham surgery or IBAT denervation. Tissues were stained with anti-TH antibody (red) and anti-perilipin antibody (green). Nuclei are blue (DAPI). Background color was set as white. Scale bar, 50 µm. All values are means ± SE. ###*P* ≤ 0.001 between surgical intervention groups; *n* = 4.

Western blot analysis showed an effective reduction in TH levels in the denervated IBAT on both diets (Fig. 5, *A* and *B*). TH levels were reduced by ∼95% in denervated animals when levels were expressed per milligram of protein in the tissue (Fig. 5*B*). By multiplying these values with total tissue protein content (from Fig. 4*D*), total levels of TH in the tissue were estimated, reflecting the total ability of the tissue to synthesize catecholamines (Fig. 5*C*).

To identify the localization of the TH protein, immunofluorescent staining of tissue sections was performed (Fig. 5*D*). As expected, TH (Fig. 5*D*, red color) localized in dotted foci within fiber-like structures. These fibers most likely correspond to sympathetic fibers, whereas the foci represent the boutons-en-passant, the contact sites between the nerves and the adipocytes (Fig. 5*D*). No major differences between chow- and HFD-fed animals were observed with regard to localization of TH (Fig. 5*D*, *top left* and *top middle left* images). Co-staining for the lipid droplet protein perilipin (green) showed the boutons-en-passant to be closely associated with adipocytes (Fig. 5*D*, *bottom left* and *bottom middle left* images). Perilipin staining also revealed the largely unilocular appearance of BAT under these conditions (long acclimation to thermoneutrality). Denervation led to a massive reduction in TH staining (Fig. 5*D*, *top right* and *top middle right* images), with very few nerve structures still being observable (not shown), probably corresponding to the 5% residual TH. Perilipin staining of denervated IBAT indicated enlarged lipid droplets and an absence of multilocular islands in the denervated IBAT of the HFD-fed animals compared with the sham-operated mice (Fig. 5*D*, *bottom images*).

#### Innervation is necessary for diet-induced recruitment of IBAT.

**Fig. 6.**
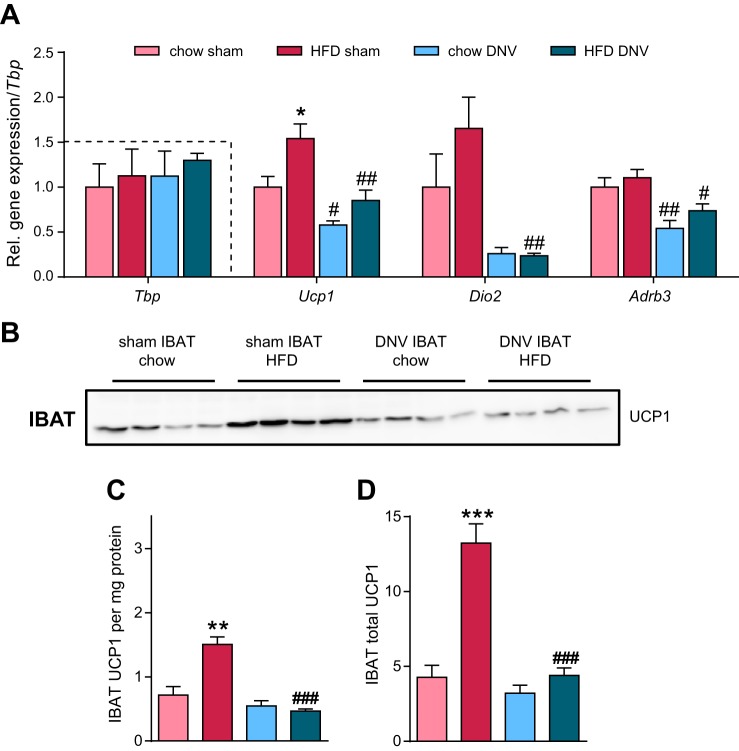
Effects of interscapular brown adipose (IBAT) denervation on IBAT recruitment. *A*: relative gene expression levels normalized to the housekeeper TATA-box binding protein (*Tbp*). Gene expression (mRNA levels) of uncoupling protein 1 (*Ucp1*), deiodinase 2 (*Dio2*), and β_3_-adrenoceptor (*Adrb3*) was measured. Dashed box indicates housekeeper levels (calculated as copy numbers, i.e., $2^{-C_{T}}$). All data were normalized to the mean of the control group (chow, sham). *B*: Western blot analysis (20 µg of total protein) of uncoupling protein 1 (UCP1) protein levels of sham-operated or IBAT-denervated (DNV) animals that were fed either chow or high-fat diet (HFD). Loading control can be seen in Fig. 5*A*. *C*: quantification of UCP1 levels per mg protein from *B*. Data in *C* and *D* are normalized to a standard IBAT sample to be able to compare UCP1 levels on different gels. *D*: IBAT total UCP1 protein (UCP1 per mg protein from *C* multiplied with total protein levels from Fig. 4*D*). This represents total UCP1 levels per depot. All values are means ± SE. *P ≤ 0.05, **P ≤ 0.01, and ***P ≤ 0.001 between diets, #*P* ≤ 0.05, ##*P* ≤ 0.01, and ###*P* ≤ 0.001 and between sham operated and denervated groups; *n* = 4–5.

Feeding mice obesogenic diets enhances the expression (mRNA levels) of the gene for UCP1 (*Ucp1*) and other thermogenic genes (91). We first analyzed the expression of thermogenic markers in the IBAT of the mice. In line with previous observations, HFD feeding increased *Ucp1* mRNA levels (Fig. 6*A*); also the mRNA levels of deiodinase 2 (*Dio2*) trended toward an increase. Denervation of IBAT reduced basal *Ucp1* mRNA levels as well as HFD-induced *Ucp1* expression (Fig. 6*A*), whereas there was a small and nonsignificant tendency toward an increase in Ucp1 gene expression in IBAT-denervated, HFD-fed animals. It should be noted that whereas *Ucp1* expression levels were indeed decreased, the expression of *Ucp1* was not zero. In line with the reduced *Ucp1* gene expression, mRNA levels of deiodinase 2 (*Dio2*) and of β_3_-adrenoceptor (*Adrb3*) were also significantly reduced in denervated IBAT on both diets (Fig. 6*A*).

Because *Ucp1* mRNA is not an estimate of total tissue thermogenic capacity (54), we also measured UCP1 protein levels (Fig. 6*B*). In agreement with earlier results (3, 20, 33, 48, 63, 85), HFD feeding increased UCP1 levels per milligram protein by approximately twofold (Fig. 6, *B* and *C*). Importantly, this HFD-induced increase in UCP1 was completely absent in denervated IBAT (Fig. 6, *B* and *C*), showing that the residual trend toward increased *Ucp1* gene expression in denervated IBAT in HFD-fed animals did not translate into higher UCP1 protein levels.

Total thermogenic capacity can best be estimated by calculating total tissue levels of UCP1 (i.e., by multiplying UCP1 levels per milligram protein from Fig. 6*C* by total protein content from Fig. 4*D*) (7, 26, 27, 84). Such analysis (Fig. 6*D*) revealed an ∼2.5-fold increase in total tissue UCP1 levels in sham-operated, HFD-fed animals compared with chow controls, which is in line with previous observations (20, 85). This increase was totally abolished in the IBAT-denervated animals (Fig. 6*D*). These findings demonstrate that innervation is essential for diet-induced recruitment of BAT thermogenic capacity (but is dispensable for basal UCP1 expression).

#### IBAT denervation does not markedly influence diet-induced recruitment in other BAT depots.

**Fig. 7.**
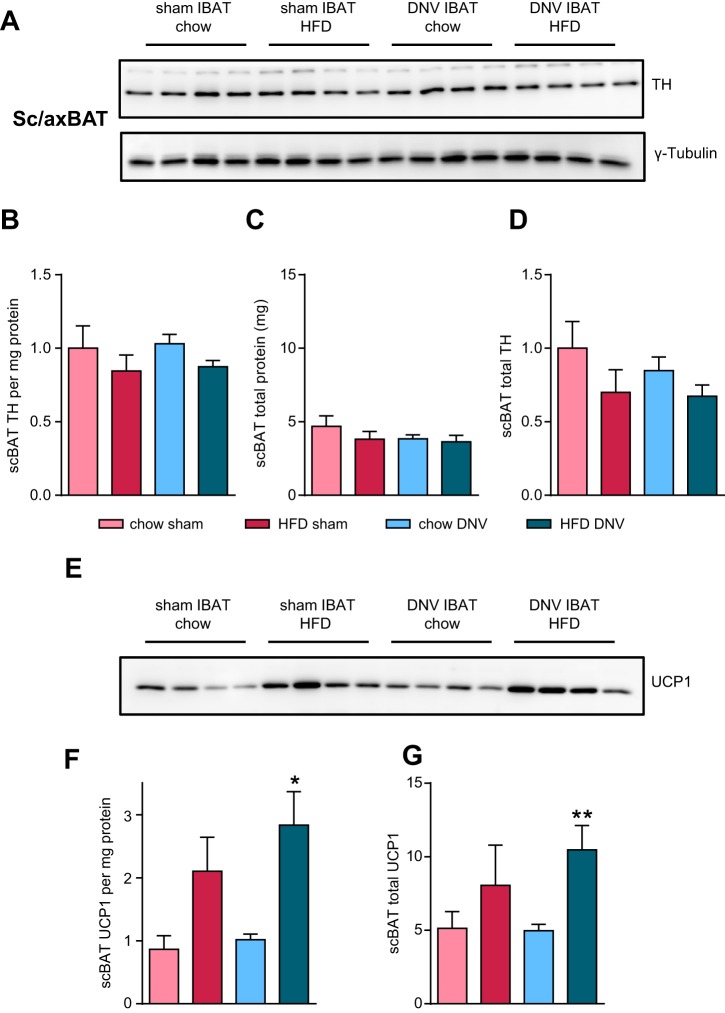
Effects of interscapular brown adipose (IBAT) denervation in another brown adipose depot. We analyzed uncoupling protein 1 (UCP1) protein levels in another brown adipose tissue (BAT) depot: the subscapular (also known as axillary) BAT depot (sc/axBAT). *A*: Western blot analysis of tyrosine hydroxylase (TH) protein levels in subscapular BAT (scBAT) of sham-operated or IBAT-denervated (DNV) animals that were fed either chow or high-fat diet (HFD). Loading control: γ-tubulin run on another gel but with the same samples. *B*: quantification of the TH levels per mg protein from *A*. *C*: total protein content of scBAT. *D*: total TH content of scBAT, calculated as data in *B*, multiplied by data in *C*, and normalized to the mean of chow-fed, sham-operated controls. *E*: Western blot analysis of UCP1 protein levels in sham-operated or IBAT-denervated (DNV) animals that were fed either chow or high-fat diet (HFD). Loading control shown in *A*. *F*: quantification of the UCP1 levels per mg protein from *E*. *G*: total UCP1 content of scBAT, calculated as UCP1 per mg protein multiplied by scBAT total protein (data in *F* multiplied by data in *C*). Data in *F* and *G* are normalized to a standard IBAT sample run on the same gel (but not shown here) to be able to directly compare UCP1 levels in different tissues. All values are means ± SE * *P* ≤ 0.05 and **P ≤ 0.01 between diets; *n* = 4.

The loss of diet-induced IBAT recruitment in denervated BAT did not lead to significant differences in body weight gain (Fig. 1*D*). To examine whether the general BAT response to HFD was still intact in other BAT depots in the IBAT-denervated mice and whether hyper-recruitment of these depots could compensate for the loss of IBAT UCP1, we measured innervation (TH) and UCP1 protein levels in another BAT depot, the subscapular (also known as axillary) BAT (scBAT). As shown in Fig. 7, *A* and *B*, the levels of tyrosine hydroxylase per milligram protein in scBAT were not affected by any of the treatments. Total protein content of scBAT was also not affected (Fig. 7*C*), and thus also the total tissue content of TH was comparable in all four groups of mice (Fig. 7*D*). As shown in Fig. 7, *E* and *F*, HFD feeding increased UCP1 levels per milligram protein in both IBAT-sham and IBAT-denervated mice. This increase was statistically robust in the IBAT-denervated animals. The total levels of UCP1 per scBAT depot were increased approximately twofold by HFD feeding in both treatment groups, although the increase was again only statistically robust in the IBAT-denervated animals (Fig. 7*G*). We thus found a tendency to hyper-recruitment (a somewhat more robust HFD-induced increase in UCP1 amount) in these other BAT depots in the IBAT-denervated mice fed an obesogenic diet, but no statistically significant differences between sham-operated and IBAT-denervated mice were observable.

#### No compensatory browning of white adipose tissue in IBAT-denervated mice.

In addition to classical brown adipocytes, another type of thermogenic adipocytes, the so-called brite (brown-like-in-white) (59) or beige (43) adipocytes exist in certain adipose depots normally considered to be white. These adipocytes are induced under certain conditions (“browning”), they are characterized by UCP1 expression, and the UCP1 is functionally thermogenic (70). The absolute contribution of brite/beige adipose tissue to whole body thermogenesis in the mouse is under debate (44, 53). However, it has been shown that denervation of IBAT under certain conditions can lead to compensatory browning of inguinal white adipose tissue (ingWAT), the most browning-competent depot (67, 88). Therefore, we analyzed innervation and UCP1 levels in ingWAT of the mice to examine the possible appearance of brite adipocytes in the white depot under conditions of diet-induced recruitment and IBAT denervation.

**Fig. 8.**
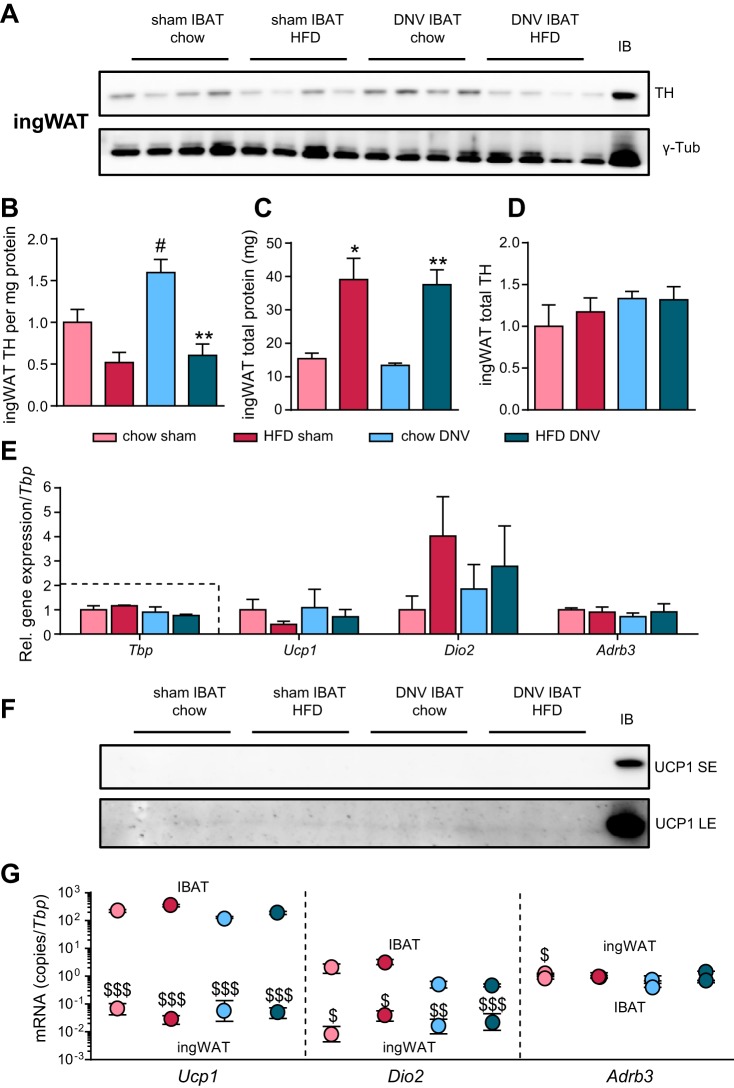
Effects of interscapular brown adipose (IBAT) denervation on browning of white adipose tissue. We analyzed the levels of uncoupling protein 1 (UCP1) in inguinal white adipose tissue (ingWAT), a browning-competent white depot, under conditions of diet-induced recruitment and IBAT denervation. *A*: Western blot analysis (20 µg of total protein) of tyrosine hydroxylase (TH) protein levels in ingWAT of sham-operated or IBAT-denervated (DNV) animals that were fed either chow or high-fat diet (HFD). Loading control: γ-tubulin run on another gel but with the same samples. *B*: quantification of the TH levels per mg protein from *A*. Significant effect of denervation: #*P* < 0.05. Significant effect of diet: **P < 0.01. *C*: total protein content of ingWAT. Significant effect of diet: *P < 0.05, **P < 0.01. *D*: total TH content of ingWAT calculated as per data in *B*, multiplied by data in *C*, and normalized to the mean of chow-fed sham-operated controls. *E*: relative gene expression levels normalized to the housekeeper TATA-box binding protein (*Tbp*). Gene expression (mRNA levels) of uncoupling protein 1 (*Ucp1*), deiodinase 2 (*Dio2*), and β_3_-adrenoceptor (*Adrb3*) was measured. Dashed box indicates housekeeper levels (calculated as copy numbers, i.e., $2^{-C_{T}}$). All data were normalized to the mean of the control group (chow, sham). *F*: Western blot analysis (20 µg of protein) of UCP1 protein levels in inguinal white adipose tissue (ingWAT) of chow- and HFD-fed mice that underwent sham operation or IBAT denervation. IB, IBAT sample (same protein amount) from chow-fed sham-operated mouse, used as positive control; LE, long image exposure; SE, short image exposure. *G*: absolute gene expression (mRNA levels) per *Tbp* of *Ucp1*, *Dio2*, and *Adrb3* in IBAT and ingWAT. Note the logarithmic scale. Average C_T_ values for the housekeeper *Tbp* in sham-operated, chow-fed controls in IBAT were 27.2 and in ingWAT 27.4. All values are means ± SE. $ *P* ≤ 0.05, $$ *P* ≤ 0.01, and $$$ *P* ≤ 0.001 between depots; *n* = 4–5.

The levels of TH per milligram of protein in ingWAT were decreased in the HFD-fed mice (Fig. 8, *A* and *B*), whereas chow-fed, IBAT-denervated mice showed increased levels of TH per milligram of protein compared with sham-operated controls (Fig. 8, *A* and *B*). At first sight, these data could be interpreted as a reflection of alterations in sympathetic stimulation of the tissue. However, because total protein content of ingWAT was strongly increased in HFD-fed mice (likely supportive tissue for the increase in total lipid content; Fig. 8*C*), the total levels of TH per ingWAT were not affected by any of the treatments (Fig. 8*D*). The observed alterations in TH-levels per milligram of protein thus appear to be mainly a reflection of TH being diluted by other proteins.

*Ucp1* expression in ingWAT was not different in any of the groups (Fig. 8*E*). No compensatory effects in the IBAT-denervated groups were observed. There was no sign of UCP1 protein expression in ingWAT of either sham-operated or IBAT-denervated mice on either diet (Fig. 8*F*). To confirm the reactivity of the antibody, an IBAT lysate from a chow-fed sham-operated animal was used as positive control. Even after long exposure (LE), there was no trace of any UCP1 protein in ingWAT under these conditions. Thus, ingWAT browning does not seem to play a role in diet-induced thermogenesis [in accordance with von Essen et al. (85)] with or without IBAT denervation.

To understand the inability to detect UCP1 protein in ingWAT, we compared the absolute gene expression levels of *Ucp1*, *Dio2*, and *Adrb3* in ingWAT and IBAT. Whereas the housekeeper levels were comparable (average C_T_ values for *Tbp* in sham-operated, chow-fed controls in IBAT were 27.2 and in ingWAT 27.4), the levels of *Ucp1* and *Dio2* mRNA expression were several orders of magnitude lower in ingWAT (note the logarithmic scale; Fig. 8*G*). C_T_ values for *Ucp1* differed by 11 units; thus the levels of *Ucp1* mRNA in ingWAT were several thousand-fold lower than in IBAT, making it understandable that it was not possible to detect UCP1 protein by Western blotting (Fig. 8*F*). Levels of *Dio2* were also orders of magnitude lower in ingWAT than in IBAT, but the differences were smaller than for *Ucp1*. The levels of *Adrb3* were comparable in IBAT and ingWAT (Fig. 8*G*).

## DISCUSSION

In the present study, we have examined the functional significance of the innervation of BAT for the adaptive response to overfeeding referred to as diet-induced thermogenesis (DIT). We used classical bilateral nerve sectioning of the interscapular depot and found that innervation was absolutely essential for the diet-induced recruitment of BAT under conditions of thermoneutrality. However, basal UCP1 levels were almost fully maintained even in the absence of innervation, and notably, other brown and white adipose depots did not markedly hyper-recruit to compensate for the absence of the activation of the interscapular depot. Several systemic effects caused by the denervation were also observed.

### 

#### Verification of denervation.

Brown adipose tissue can be denervated surgically or chemically, and differences in response between the methods have been observed. Chemical denervation with 6-OH-dopamine is selective for the sympathetic nerves but not fully reliable for full sympathectomy in BAT (16, 68, 77, 80). Here, we selected surgical denervation as being fully efficient to produce sympathectomy. However, in this process, we would necessarily also cut nonsympathetic nerves, any other efferent nerves, and afferent sensory nerves running in the same nerve bundles (4, 15, 35, 36, 40, 64, 79, 80, 86), and through this we may possibly have affected systemic events.

Although it is unlikely that cross-innervation exists between the two lateral interscapular brown adipose tissue depots (86), we elected to make bilateral nerve sectioning to avoid possible problems due to such cross-innervation. We cut the nerves at two locations and also removed the nerve between the sections to prevent regrowth. We found that the procedure was successful in bringing about localized sympathectomy in that tyrosine hydroxylase was eliminated to 95% from the interscapular depot (Fig. 5*C*), whereas the tyrosine hydroxylase was fully preserved in a nondenervated BAT depot (Fig. 7*A*) and in ingWAT (Fig. 8*A*).

#### A high-fat diet cannot induce UCP1 in the IBAT of IBAT-denervated mice.

**Fig. 9.**
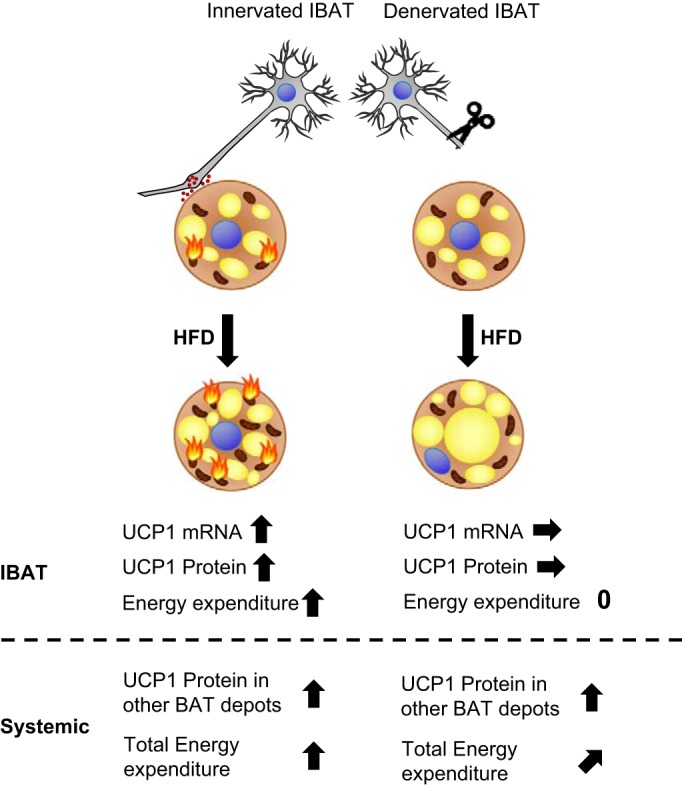
Model. Sympathetic innervation is essential for diet-induced recruitment of brown adipose tissue. Under conditions of normal brown adipose tissue (BAT) innervation (*left*), high-fat diet (HFD) feeding activates the sympathetic nervous system, leading to the release of norepinephrine (small red dots) that activates β-adrenoceptors on brown adipocytes, thereby activating uncoupling protein 1 (UCP1) gene expression, leading with time to an increased UCP1 amount (indicated by increased number of mitochondria) and through this increasing the capacity and ongoing rate of metabolism (indicated by flames). If sympathetic signaling is abolished by denervation (*right*), diet-induced recruitment of BAT does not take place (no increase in mitochondria; no flames), whereas lipid accumulation is increased. However, basal UCP1 levels are not reduced. Systemically, in both interscapular brown adipose (IBAT)-denervated and sham-operated mice, other BAT depots are also recruited in response to HFD feeding. However, the total energy expenditure response to HFD is halved in IBAT-denervated mice compared with sham-operated controls, which roughly reflects the estimated contribution of IBAT to total BAT depots. Gray, neurons; small red dots, norepinephrine; dark brown, mitochondria; yellow, lipid droplets; blue, nuclei; flames, thermogenesis.

We found that in sham-operated mice, high-fat diet feeding led to more than a doubling of the content of UCP1 in the interscapular BAT (Figs. 6*D* and Fig. 9), in principle in agreement with expectations based on earlier observations (3, 20, 48, 61, 63, 85). However, in the denervated mice, there was no such effect of this diet (Fig. 6*D*). Thus, it must be concluded that the presence of an intact nervous system supply to the tissue is at least absolutely essential for the recruitment process associated with DIT (Fig. 9). A very recent study with another feeding model arrived at a similar conclusion (51). We find it likely that the nervous system is not only necessary for recruitment of BAT but that it is indeed neuronal stimulation that induces and regulates the recruitment process, but this principally cannot be concluded from these type of experiments. The observation that feeding rats a high-sucrose diet stimulates sympathetic nerve activity (92) strengthens the idea that the sympathetic nervous system is the main mediator of the recruitment response to obesogenic diets.

Diet-induced brown fat recruitment is thus analogous to cold-induced brown fat recruitment, where ample evidence exists for the essentiality of the nervous system for recruitment (17, 18, 21, 34, 45, 60). Thus, both established physiological functions of the tissue are fully dependent on innervation.

Although it cannot be excluded based on the present experiments that novel, nonsympathetic brown fat activators (82, 83) are determinative for the diet-induced recruitment observed here (and that the present experiments would thus only define a necessary but supportive role for the innervation), it can minimally be concluded that the novel activators are themselves unable to induce brown fat recruitment. Rather, such factors would activate the sympathetic nervous system, which then in turn activates thermogenesis. One of the most prominent candidates would be the adipokine leptin, which is increased in the obese state (32) and has been described to activate brown adipose tissue sympathetic activity (8) and thermogenesis (9, 10, 65). However, we have earlier found no evidence to support such a thermogenic function of leptin (23, 26), and leptin-deficient *ob/ob* mice still display diet-induced recruitment of BAT (41), making it unlikely that leptin is the mediator of diet-induced thermogenesis. Because cold-induced and diet-induced thermogenesis are the only widely accepted physiological functions of brown adipose tissue, and since the novel activators cannot in either of these functions (in absence of innervation) by themselves elicit the adaptive process, the physiological significance of the novel activators has yet to be elucidated (but see below).

#### A basal level of UCP1 expression is independent of innervation.

The mice maintained a low amount of UCP1 in IBAT even under the physiological conditions encountered here: chow feeding and thermoneutrality (Figs. 6*D* and 9). Even this level of UCP1 is sufficient to mediate thermogenesis, at least in isolated brown fat mitochondria (69). The presence of this amount of UCP1 is in itself noteworthy. This is because in lean, chow-fed, thermally comfortable mice, there is no obvious demand for extra thermogenesis, and the functional role of the thermogenically competent tissue is thus not easily comprehended. Additionally, the presence of low amounts of UCP1 is not an inherent property of the brown fat cells. In cell cultures of primary brown adipocytes, the expression level of UCP1 is practically nonexistent in the absence of adrenergic or other stimulation (58). Thus, the presence of UCP1 in the brown adipose tissue in the mouse implies the existence of an external factor inducing a low but functional stimulation of the tissue. Thus, the low expression of UCP1 could have been explainable by a low tone of the sympathetic nervous system. However, we found (Fig. 6*D*) that denervation of the tissue in the chow-fed mice had almost no effect on the UCP1 content (Fig. 9). Thus, a weak adrenergic drive is not the cause of the UCP1 gene expression, nor can it result from any other substance released from the nervous supply. These observations are principally in agreement with earlier observations under comparable conditions where denervation led to lower UCP1 levels, but they were not reduced to zero (17, 18, 21, 38, 45). Also, mice with defective catecholamine synthesis still have detectable basal levels of UCP1 expression (76), strengthening the idea that nonsympathetic (nonneuronal) mechanisms contribute to maintenance of basal UCP1 expression.

Thus, under these nonadrenergically stimulated conditions, a role could exist for nonadrenergic factors, such as those discussed previously (66, 82, 83). The factors include endogenous PPARγ agonists; exogenous PPARγ agonists are able to induce brown fat recruitment not only in innervated tissue (21, 22, 30) but to a lower degree even in denervated tissue (21). Our results clearly establish, however, that this basal level of UCP1 could not be further recruited by such nonnervous system factors.

#### No marked compensatory recruitment of other adipose depots.

Although the IBAT depot under these conditions would normally only represent ∼45% of the thermogenic capacity of brown adipose tissue, the virtual absence of effect of the IBAT denervation on the development of body weight/obesity in HFD-fed mice (Figs. 1*C* and 4*A*) could imply that hyper-recruitment of other BAT or WAT depots compensated for the absence of increased UCP1 amounts in IBAT. Denervation of BAT has been described to trigger browning of white adipose tissue under conditions of active thermogenesis (67, 88), and surgical removal of interscapular brown adipose tissue may (11, 73) or may not (12) lead to hyper-recruitment of other depots. In mice overexpressing *Cidea* in adipose tissues, leading to lowered thermogenic competence, this lowered competence is compensated by increased recruitment of the tissue to match the cold-induced thermogenic demand of the animal (27). Thus, hyper-recruitment may be expected to be induced by the denervation. However, we did not find significant evidence for increased recruitment of another BAT depot in our HFD-fed, IBAT-denervated mice. It cannot be excluded that other BAT depots reacted more strongly to the IBAT denervation, so whole body UCP1 levels might still be comparable in the sham-operated and IBAT-denervated mice; however, the scBAT depot examined is the second largest of the classical BAT depots (14). Thus, there is clearly not a strong general hyper-recruitment of all other BAT depots.

Additionally, no browning of inguinal white adipose tissue was detectable in any of the groups. The reason for this may lie in the fact that browning of white adipose tissue requires stronger stimuli (e.g., cold adaptation) than that needed for the induction of UCP1 in BAT (44), and HFD-feeding by itself does not lead to browning of ingWAT under thermoneutral conditions (Fig. 8) (85).

#### Systemic effects of IBAT denervation.

In addition to the effects of IBAT denervation on IBAT itself and on other adipose tissues discussed above, the denervation also led to a series of both expected and unexpected systemic effects, as detailed below.

#### An alteration in diurnal feeding pattern.

The mice given chow consumed around one-third of their food during daytime (Fig. 2*A*). As expected, the mice given the high-fat diet ate more than those given the chow diet. This was accompanied by increased meal frequency in these mice. We observed that this overconsumption, at least in the metabolic chambers, was largely explained by the mice also eating during daytime (Fig. 2*B*), in agreement with Kohsaka et al. (47) and Pendergast et al. (57). Because these mice had been given the high-fat diet for 5 wk before the metabolic chamber experiment, the daytime eating cannot be ascribed to the “excitement” value of the high-fat diet.

An unexpected effect of denervation of IBAT was a change in the onset time of nocturnal eating. In normal mice, nocturnal eating commences some hours before lights are turned off – but in the IBAT-denervated mice, it did not commence until the lights went out (easily observable in the chow-fed mice and discernible in the high-fat diet-fed mice; Fig. 2, *A* and *B*). This was actually the only observable effect of denervation in the chow-fed mice, and the shift in food intake pattern was not accompanied by a similar shift in the diurnal pattern of energy expenditure (Fig. 3, *A* and *B*). From the present study, there is thus some indication that the interscapular BAT depot possesses properties in addition to its pure thermogenic capacity that allow it to substantially influence metabolism, but the relationship between IBAT innervation and diurnal food intake pattern is not readily understandable. A hypothesis that heat generated from BAT could be a signal for meal cessation has been forwarded earlier (37, 39, 50); no heat is generated from the denervated IBAT, and this could perhaps in some way affect meal patterns.

#### No increased body weight gain.

Denervation of IBAT did not lead to increased weight gain on chow or high-fat diet (Figs. 1*C* and 4*A*). The recruitment of BAT normally encountered when animals are exposed to an obesogenic diet is expected to partially counteract the development of obesity. Because the IBAT represents ∼45% of the total thermogenic capacity of total BAT, a tendency to an increased body weight gain would have been expected in the IBAT-denervated mice, with everything else being unaltered, but this was not seen.

#### A change in food intake and metabolic efficiency.

Although only the IBAT depot was denervated, an effect on total food intake was observed in the high-fat diet-fed mice (Fig. 1*E* and particularly Fig. 2*D*). The IBAT-denervated mice seemed to compensate for the decreased thermogenesis in IBAT by decreasing food intake, an effect observed earlier in rats (13). An explanation for this could be that the central mechanism for body weight control compensated for the reduced brown-fat-derived energy utilization by reducing food intake. Decreased food intake, together with an unchanged body weight increase, results in an increased metabolic efficiency (Fig. 1*F*), implying a significant effect of the IBAT fraction of the total brown adipose depots (even under conditions of relatively low expression of UCP1) on total energy expenditure.

#### A lower ability to respond thermogenically to a high-fat diet.

The clearest systemic effect of IBAT denervation was observed as a reduced ability of the mice to actively demonstrate diet-induced thermogenesis. Thus, in sham-operated mice, chronic exposure to a high-fat diet led to a clear increase in daily energy expenditure (Fig. 3*C*). In the denervated mice, this increase was halved (Figs. 3*D* and 9 and Table 1). The effect of denervation of the interscapular depot thus seems to almost be proportional to that expected from the contribution of the depot to diet-induced thermogenesis, as estimated from its relative UCP1 content (44).

### Conclusions and Perspectives

In the present investigation, we demonstrate that the increase in thermogenic capacity observed under conditions of chronic overeating and obesity (diet-induced thermogenesis) is fully dependent on innervation of the brown adipose tissue depots. Therefore, in this respect, diet-induced recruitment is analogous to cold-induced recruitment. In both cases, it is clear that nonneuronal blood-borne factors in themselves cannot recruit brown adipose tissue thermogenic capacity but that the nervous stimulation of the tissue is essential and likely determinative for the recruitment processes in the tissue. Thus, to be able to utilize brown adipose tissue thermogenic capacity to alter the balance between food intake and energy storage, this study points distinctly to the necessity to understand the physiological mechanisms that transfer food intake and obesity into a central signal that is transmitted via the sympathetic nervous system to the brown adipose tissue.

## GRANTS

This study was supported by grants from the Swedish Research Council, the German Research Council (DFG-HE3645/7-1), and the Studienstiftung des deutschen Volkes.

## DISCLOSURES

No conflicts of interest, financial or otherwise, are declared by the authors.

## AUTHOR CONTRIBUTIONS

J.N. conceived and designed research; A.W.F. and C.S. performed experiments; A.W.F., C.S., and J.N. analyzed data; A.W.F., C.S., B.C., J.H., and J.N. interpreted results of experiments; A.W.F. prepared figures; A.W.F. drafted manuscript; A.W.F., B.C., J.H., and J.N. edited and revised manuscript; A.W.F., C.S., B.C., J.H., and J.N. approved final version of manuscript.
